# Facilitation of Long-Term Potentiation by Muscarinic M_1_ Receptors Is Mediated by Inhibition of SK Channels

**DOI:** 10.1016/j.neuron.2010.11.018

**Published:** 2010-12-09

**Authors:** Katherine A. Buchanan, Milos M. Petrovic, Sophie E.L. Chamberlain, Neil V. Marrion, Jack R. Mellor

**Affiliations:** 1 Medical Research Council Centre for Synaptic Plasticity, School of Physiology and Pharmacology, University of Bristol, University Walk, Bristol BS8 1TD, UK; 2 Department of Neuroscience, Physiology and Pharmacology, University College London, London WC1E 6BT, UK

## Abstract

Muscarinic receptor activation facilitates the induction of synaptic plasticity and enhances cognitive function. However, the specific muscarinic receptor subtype involved and the critical intracellular signaling pathways engaged have remained controversial. Here, we show that the recently discovered highly selective allosteric M_1_ receptor agonist 77-LH-28-1 facilitates long-term potentiation (LTP) induced by theta burst stimulation at Schaffer collateral synapses in the hippocampus. Similarly, release of acetylcholine by stimulation of cholinergic fibers facilitates LTP via activation of M_1_ receptors. N-methyl-D-aspartate receptor (NMDAR) opening during theta burst stimulation was enhanced by M_1_ receptor activation, indicating this is the mechanism for LTP facilitation. M_1_ receptors were found to enhance NMDAR activation by inhibiting SK channels that otherwise act to hyperpolarize postsynaptic spines and inhibit NMDAR opening. Thus, we describe a mechanism where M_1_ receptor activation inhibits SK channels, allowing enhanced NMDAR activity and leading to a facilitation of LTP induction in the hippocampus.

## Highlights

► The muscarinic M1 receptor allosteric agonist 77-LH-28-1 facilitates LTP induction ► M1 receptor activation inhibits SK channel function ► Inhibition of SK channels enhances NMDA receptor opening during LTP induction ► PKC mediates the inhibition of SK channels by M1 receptors

## Introduction

The cholinergic system in the brain plays a major role in learning and memory through activation of muscarinic acetylcholine receptors (mAChRs). Antagonists of mAChRs, such as scopolamine, impair the encoding of new memories in animal models of learning and memory (De Rosa and Hasselmo, 2000; Warburton et al., 2003) and produce cognitive impairment in humans (Atri et al., 2004). mAChR agonists and antagonists also modulate the induction of synaptic plasticity in the hippocampus, a cellular correlate for learning and memory. Both the endogenous release of acetylcholine in vivo (Leung et al., 2003; Ovsepian et al., 2004) and the exogenous application of mAChR agonists in vitro facilitate the induction of long-term potentiation (LTP) (Boddeke et al., 1992; Shimoshige et al., 1997; Shinoe et al., 2005). It is, therefore, tempting to infer that activation of mAChRs by release of acetylcholine in the hippocampus facilitates the induction of synaptic plasticity leading to cognitive enhancement.

Of the five mAChR subtypes potentially involved in cognitive enhancement, the M_1_ subtype has received much attention because of its ubiquitous expression in the cortex and hippocampus. Learning, working memory, and the induction of synaptic plasticity are all impaired in M_1_ receptor knockout mice (Anagnostaras et al., 2003; Shinoe et al., 2005; Wess, 2004). Furthermore, putative M_1_ receptor-specific agonists improve cognitive function in animal models (Dean et al., 2003) and facilitate LTP induction (Boddeke et al., 1992; Seol et al., 2007) although the selectivity of these agonists remains unclear. A new group of allosteric agonists potentially offers greater subtype specificity, e.g., AC-42 at M_1_ receptors (Langmead et al., 2006) and LY2033298 at M_4_ receptors (Chan et al., 2008). An AC-42 derivative, 77-LH-28-1, has subsequently been developed that penetrates the brain and exhibits full agonist activity at M_1_ receptors (Langmead et al., 2008).

mAChR activation could facilitate LTP induction by direct enhancement of N-methyl-D-aspartate (NMDA) receptor (NMDAR) opening (Aramakis et al., 1999; Harvey et al., 1993; Marino et al., 1998; Markram and Segal, 1992) and/or reduced attenuation of back-propagating action potentials (Tsubokawa and Ross, 1997). mAChRs also inhibit potassium channels, such as KCNQ channels, which results in an increased input resistance. In addition, recent evidence suggests that ion channels at postsynaptic spines work in unison with neurotransmitter receptors, enzymes, and other protein partners, thus creating multiprotein functional units (Allen et al., 2007; Bildl et al., 2004). One such example is the small conductance voltage-independent and calcium-dependent SK channels that form feedback loops with NMDARs and ultimately shape excitatory post-synaptic potentials (EPSPs) and the induction of LTP (Behnisch and Reymann, 1998; Bloodgood and Sabatini, 2007; Faber, 2010; Faber et al., 2005; Gu et al., 2008; Ngo-Anh et al., 2005).

Here, we find that the specific M_1_ receptor agonist 77-LH-28-1 facilitates LTP at the Schaffer collateral synapse. Importantly, our data indicate that M_1_ activation leads to a previously unknown dynamic regulation of SK channel activity and subsequent modulation of NMDAR opening in response to synaptic activation. We conclude that the inhibition of SK channels is a critical link between M_1_ receptor activation and the facilitation of LTP.

## Results

### The Cellular and Synaptic Effects of M_1_ Receptor Activation

To study the role of the M_1_ receptor in synaptic plasticity, we made use of the recently discovered selective M_1_ receptor agonist 77-LH-28-1 (1-[3-(4-butyl-1-piperidinyl)propyl]-3,4-dihydro-2[1H]-quinolinone). This compound is an allosteric agonist exhibiting >100-fold specificity for M_1_ over other mAChR subtypes. We have previously shown that 10 μM 77-LH-28-1 selectively activates M_1_ receptors in cell lines expressing specific human mAChRs and in rat hippocampal slices (Langmead et al., 2008).

**Figure 1:**
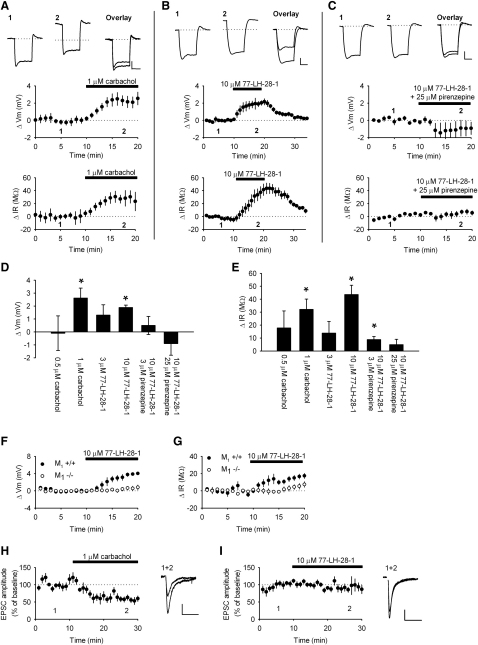
The Effect of Cholinergic Agonists on the Cellular and Synaptic Properties of CA1 Pyramidal Cells (A) Bath application of 1 μM carbachol caused a depolarization of the membrane potential (middle) and an increase in input resistance (bottom). Top: Sample voltage traces from a single experiment demonstrating the voltage response to a 100 pA current step during baseline (1) and in the presence of 1 μM carbachol (2). The dotted line represents the baseline membrane potential and the overlay shows the offset traces. (B) Bath application of 10 μM 77-LH-28-1 caused a depolarization of the membrane potential (middle) and increase in input resistance (bottom) that reversed on washout. Top: Sample voltage traces as described in (A). (C) The effect of 10 μM 77-LH-28-1 on the membrane potential (middle) and input resistance (bottom) of CA1 pyramidal cells was blocked by the coapplication of 25 μM pirenzepine (black bar). Top: Sample traces as described in (A). The scale bars represent 2 mV, 100 ms. Summary bar graph of the concentration-dependent membrane potential (D) or input resistance (E) changes 8–10 min after application of cholinergic agonists and antagonists. ^∗^ indicates a significant difference from baseline values in (D) and (E) (p < 0.05). Bath application of 10 μM 77-LH-28-1 caused a depolarization of the membrane potential (F) and an increase in input resistance (G) in slices taken from M_1_+/+ mice but not M_1_−/− mice. (H) Bath application of 1 μM carbachol caused a depression of the evoked EPSC amplitude. Right: Sample EPSC traces from a single experiment during baseline and in the presence of 1 μM carbachol. The scale bars represent 10 pA, 20 ms. (I) Bath application of 10 μM 77-LH-28-1 resulted in no change in EPSC amplitude. Right: Sample EPSC traces as described in (H). The scale bars represent 20 pA, 20 ms. The data are plotted as the mean ± SEM.

We first characterized the effects of 77-LH-28-1 on CA1 pyramidal cells examining both membrane properties and glutamatergic synaptic transmission in the Schaffer collateral pathway. These actions were compared to those of the nonselective cholinergic agonist carbachol. Whole-cell patch clamp recordings were made from visually identified CA1 pyramidal neurons in hippocampal slices. In current clamp mode, bath application of 1 μM carbachol caused a depolarization of 2.6 ± 0.8 mV (Figures 1A and 1D; n = 8, p < 0.05) and elicited an increase in the input resistance of 32 ± 8 MΩ (Figures 1A and 1E; 206 ± 20 MΩ to 234 ± 15 MΩ, n = 8, p < 0.05). The mean resting membrane potential was −75 ± 1 mV (n = 15). A similar depolarization and increase in input resistance was seen with bath application of 10 μM 77-LH-28-1 that reversed with washout (Figures 1B, 1D, and 1E; 1.9 ± 0.2 mV, p < 0.05, and 44 ± 7 MΩ, p < 0.05, n = 6). The depolarization and increase in input resistance were concentration-dependent for both carbachol and 77-LH-28-1. Application of 0.5 μM carbachol failed to produce a statistically significant depolarization of the membrane potential (Figure 1D; −0.1 ± 1.3 mV, n = 6, p > 0.05) or increase in input resistance (Figure 1E; 18 ± 13 MΩ, n = 6, p > 0.05), and 3 μM 77-LH-28-1 also failed to significantly depolarize the membrane potential (Figure 1D; 1.3 ± 0.8 mV, n = 5, p > 0.05) or produce a significant increase in input resistance (Figure 1E; 14 ± 9 MΩ, n = 5, p > 0.05).

The effects of 77-LH-28-1 on the cellular properties of CA1 pyramidal cells were blocked by addition of the M_1_ receptor antagonist pirenzepine, again in a concentration-dependent manner. Application of 3 μM pirenzepine reduced the depolarization induced by 10 μM 77-LH-28-1 to 0.5 ± 0.7 mV (Figure 1D; n = 8, p > 0.05) and the increase in input resistance to 9 ± 2 MΩ (Figure 1E; n = 8, p < 0.05). Application of 25 μM pirenzepine reduced the depolarization to −1 ± 1 mV and the increase in input resistance to 5 ± 4 MΩ (Figures 1C, 1D, and 1E; n = 4, p > 0.05). The effects of carbachol and 77-LH-28-1 on the cellular properties of CA1 pyramidal cells are therefore similar.

The specificity of 77-LH-28-1 was verified in mice lacking the M_1_ receptor. Application of 10 μM 77-LH-28-1 produced a 3.9 ± 3 mV depolarization of the membrane potential in slices prepared from M_1_+/+ mice (n = 5, p < 0.05) but no depolarization in slices from M_1_−/− mice (Figure 1F; 0.7 ± 0.5 mV, n = 8, p > 0.05). The mean resting membrane potential in cells from either set of animals prior to agonist application was not significantly different (−70 ± 2 mV and −71 ± 1 mV for +/+ and −/− mice, respectively, p > 0.05). Similarly, 77-LH-28-1 produced a 16 ± 3 MΩ increase in input resistance in slices prepared from M_1_+/+ mice (n = 6, p < 0.05) that was abolished in slices from M_1_−/− mice (Figure 1G; 6 ± 3 MΩ, n = 8, p > 0.05). These results further illustrate the specificity of 77-LH-28-1 for the M_1_ receptor.

It is known that activation of cholinergic receptors in hippocampal slices induces short- and long-term changes in synaptic transmission, depending on the precise recording conditions and agonist used (Auerbach and Segal, 1996; Dickinson et al., 2009; Fernández de Sevilla et al., 2008; Markram and Segal, 1990; Shimoshige et al., 1997). This has been attributed by various groups to activation of nicotinic or muscarinic receptors. Therefore, we next examined the effects of muscarinic receptor activation on synaptic transmission. By maintaining CA1 pyramidal cells in voltage clamp and recording excitatory postsynaptic currents (EPSCs) in response to stimulation of Schaffer collateral axons, we found that application of 1 μM carbachol induced a depression in EPSC amplitude (Figure 1H; 58% ± 6% of baseline 15–20 min after application, n = 5, p < 0.05). If the concentration of carbachol was raised to 5 μM, the EPSC depression increased (41% ± 9% of baseline, n = 5, p < 0.05, data not shown), and lowering it to 0.5 μM decreased the depression (71% ± 9%, n = 7, p < 0.05, data not shown). In contrast, 10 μM 77-LH-28-1 had no significant effect on EPSCs (Figure 1I; 81% ± 10% of baseline 15–20 min after application, n = 7, p > 0.05) while still increasing the input resistance of the postsynaptic cell (31 ± 11 MΩ, n = 7, p < 0.05). This indicates that the depolarizing action of acetylcholine is due to M_1_ receptor activation, but the effects on synaptic transmission are due to activation of nicotinic receptors or mAChRs other than M_1_. The specificity of 77-LH-28-1 enabled us to examine the role of M_1_ receptors in synaptic plasticity since 77-LH-28-1 had limited effects on baseline synaptic transmission.

### M_1_ Receptors Facilitate Induction of LTP in the Hippocampus

**Figure 2:**
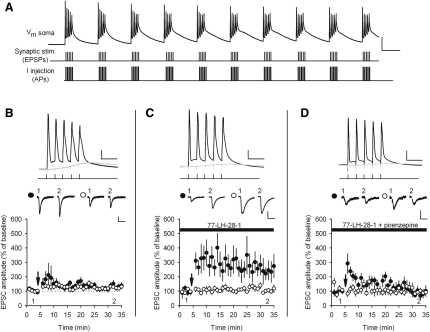
The M_1_ Receptor Agonist 77-LH-28-1 Facilitates the Induction of LTP by TBP (A) Diagram of TBP protocol. Top: Voltage trace of TBP protocol recorded at the soma of a CA1 pyramidal cell. The scale bars represent 40 mV, 100 ms. Middle and bottom: Traces illustrate the timing of inputs to the stimulating and recording electrodes evoking EPSPs and somatic action potentials, respectively. (B) TBP does not induce LTP under control conditions. Bottom: Coincident TBP of subthreshold EPSPs and somatic action potentials induced no change in EPSC amplitude in the test (black circles) or control (white circles) pathways. The arrow indicates the timing of the TBP protocol. Top: Example of voltage traces from a single experiment showing the initial burst of five coincident EPSPs and action potentials (black) and a single test burst of five subthreshold EPSPs (gray). The scale bars represent 20 mV, 20 ms. Middle: Example of current traces from a single experiment illustrating the mean EPSC response during the baseline (1) and at 30–35 min (2) in the test and control pathways. The scale bars represent 10 pA, 40 ms. (C) TBP does induce LTP in the presence of the M_1_ receptor agonist 77-LH-28-1. Bottom: In the presence of 77-LH-28-1 (10 μM), coincident TBP of subthreshold EPSPs and somatic action potentials induced pathway-specific LTP. Symbols as described in (B). Top: Example of voltage traces as described in (B). The scale bars represent 20 mV, 20 ms. Middle: Example of EPSC current traces from a single experiment as described in (B). The scale bars represent 20 pA, 40 ms. (D) TBP does not induce LTP in the presence of the M_1_ receptor agonist 77-LH-28-1 and the M_1_ receptor antagonist pirenzepine. Bottom: Coapplication of pirenzepine (25 μM) and 77-LH-28-1 (10 μM) prevented the induction of pathway-specific LTP. Symbols as described in (B). Top: Example of voltage traces as described in (B). The scale bars represent 20 mV, 20 ms. Middle: Example of EPSC current traces from a single experiment as described in (B). The scale bars represent 10 pA, 40 ms. The data are plotted as the mean ± SEM.

We next investigated the role of selective M_1_ receptor activation on LTP induced by a theta burst pairing (TBP) protocol. We have previously shown this protocol does not induce LTP when EPSP amplitude is kept below threshold for the initiation of action potentials but can induce LTP when EPSPs are suprathreshold (Buchanan and Mellor, 2007), indicating that TBP with subthreshold EPSP amplitudes is just below the threshold for LTP induction. In the experiments described here, synaptic strength was recorded in voltage clamp in two independent Schaffer collateral pathways. In current clamp, TBP was then applied by pairing stimulation to one of the input pathways with initiation of back-propagating action potentials (b-APs, 2 ms current injections of amplitude 2 nA) in the postsynaptic cell. Recordings were then returned to voltage clamp mode to measure the EPSC amplitude in the two input pathways. Importantly, two input pathways were used in all plasticity experiments to ensure that the application of cholinergic agonists did not cause any nonspecific changes in synaptic strength. In addition, in all experiments, baseline EPSC amplitudes were small (mean 10 ± 3 pA, n = 12) to avoid suprathreshold summation of EPSPs during TBP (mean EPSP summation 6.1 ± 1.4 mV, mean resting potential −71 ± 2 mV, n = 12) (Figure 2A; see Experimental Procedures for full description of induction protocol). In agreement with our previous results, no LTP was induced by TBP (Figure 2B; 126% ± 10% control versus 136% ± 20% test pathway, n = 12, p > 0.05). In contrast, in the presence of 10 μM 77-LH-28-1, robust pathway-specific LTP was induced (Figure 2C; 105% ± 16% control versus 243% ± 56% test pathway, n = 7, p < 0.05). The action of 77-LH-28-1 was specific for M_1_ receptors because addition of pirenzepine (25 μM) prevented the induction of LTP (Figure 2D; 91% ± 17% control versus 85% ± 16% test pathway, n = 7, p > 0.05).

The experiments were repeated at near physiological temperatures (35°C) to check that the effects were not specific to room temperature conditions. In control conditions, TBP did not produce a significant increase in EPSC amplitude (Figure S1A, available online; 103% ± 13% control versus 107% ± 17% test pathway, n = 7, p > 0.05). In the presence of 10 μM 77-LH-28-1, LTP was similar in amplitude to that found at room temperature (Figure S1B; 94% ± 4% control versus 358% ± 34% test pathway, n = 8, p < 0.01).

We also investigated the facilitation of LTP by 77-LH-28-1 using field potential recordings from hippocampal slices. Stimulation of the Schaffer collateral pathway with a theta burst protocol, consisting of 10 bursts at a frequency of 5 Hz where each burst consisted of five pulses at 100 Hz, produced a small but significant LTP (Figure S1C; 113% ± 4%, n = 7, p < 0.05). In the presence of 77-LH-28-1, the amount of LTP was increased to 135% ± 7% (n = 6, p < 0.05), demonstrating that M_1_ receptor activation facilitates LTP in agreement with previous reports (Ovsepian et al., 2004; Shinoe et al., 2005). When a maximal LTP protocol was employed, consisting of the theta burst protocol repeated three times with an interval of 10 s, this induced a larger LTP, and M_1_ receptor activation was unable to facilitate LTP (Figure S1D; 133% ± 11% control versus 137% ± 5% 77-LH-28-1, n = 6 and 6).

**Figure 3:**
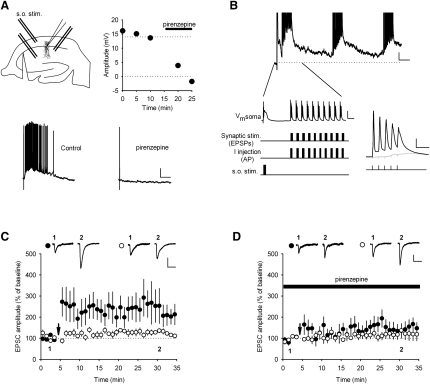
The Endogenous Release of Acetylcholine Acting at M_1_ Receptors Facilitates the Induction of LTP by TBP (A) mAChR-mediated EPSPs can be induced in hippocampal slices. Top left: Schematic of a hippocampal slice demonstrating the positioning of the stimulating electrodes in stratum radiatum and stratum oriens (s.o.). Top right: Example of experiment shows the slow EPSP evoked by stratum oriens stimulation was blocked by the M_1_ receptor antagonist pirenzepine (25 μM). Bottom: Example of voltage traces of the slow EPSP in control conditions and in the presence of pirenzepine (25 μM). The scale bars represent 10 mV, 2 s. (B) Illustration of mAChR-mediated EPSP and TBP protocol. Top: Example of voltage trace of the TBP protocol recorded at the soma during stimulation of the slow mAChR-mediated EPSP. The scale bars represent 2 mV, 2 s. Bottom Left: Schematic of the initial train of the TBP protocol illustrating the timing of the TBP in relation to stratum oriens stimulation. The scale bars represent 20 mV, 200 ms. Bottom right: Example of voltage trace from a single experiment showing the initial burst of five coincident EPSPs and action potentials (black) and a single test burst of five subthreshold EPSPs (gray) applied before stratum oriens stimulation. The scale bars represent 20 mV, 20 ms. (C) TBP does induce test pathway-specific LTP with simultaneous stimulation of the muscarinic EPSP. Arrow indicates the timing of the concurrent stratum oriens stimulation and TBP. Top: Example of EPSC current traces from a single experiment illustrating the mean EPSC response during the baseline (1) and at 30–35 min (2) in the test (black circles) and control (white circles) pathways. The scale bars represent 20 pA, 40 ms. (D) The M_1_ receptor antagonist pirenzepine (25 μM) prevented the induction of LTP by concurrent stratum oriens stimulation and TBP. Symbols as described in (C). Top: Example of EPSC current traces from a single experiment as described in (C). The scale bars represent 10 pA, 40 ms. The data are plotted as the mean ± SEM.

We next tested whether the release of endogenous acetylcholine from cholinergic fibers in the hippocampus could activate M_1_ receptors on CA1 pyramidal cells and facilitate the induction of LTP. Stimulation of cholinergic fibers in stratum oriens (four stimuli at 100 Hz) in the presence of the glutamatergic and GABAergic antagonists NBQX (1 μM), D-AP5 (50 μM), LY341495 (100 μM), picrotoxin (50 μM), and CGP55845 (1 μM) resulted in long-lasting mAChR-mediated EPSPs that were similar in amplitude and duration to those seen by other researchers (Figure 3A) (Cole and Nicoll, 1983; Shinoe et al., 2005). mAChR-mediated EPSPs were completely blocked by pirenzepine (25 μM), demonstrating the role of M_1_ receptors (Figure 3A). The average amplitude and duration of mAChR-mediated EPSPs was 24 ± 4 mV and 19 ± 1 s (n = 5, pirenzepine blocked on average 100% ± 2% of the response). To test the role of the mAChR-mediated EPSP in gating LTP, we omitted the glutamatergic and GABA_B_ receptor antagonists and stimulated the stratum oriens 2 s before TBP to ensure that the mAChR-mediated EPSP was maximal during the induction protocol (Figure 3B). Stimulation of the stratum oriens enabled the induction of substantial pathway-specific LTP (Figure 3C; 121% ± 8% control versus 208% ± 44% test pathway, n = 15, p < 0.05), and this was shown to result from M_1_ receptor activation because the effect was blocked by bath application of pirenzepine (Figure 3D; 123% ± 30% control versus 149% ± 31% test pathway, n = 8, p > 0.05). The average peak amplitude of the mAChR-mediated EPSP during the LTP induction protocol was 5.0 ± 1.5 mV with a range of 1 to 22 mV. However, there was no correlation between the amount of LTP induced and the amplitude of the mAChR-mediated EPSP during LTP induction (data not shown).

### M_1_ Receptor Enhancement of NMDAR-Mediated EPSCs Is Indirect and Voltage Dependent

**Figure 4:**
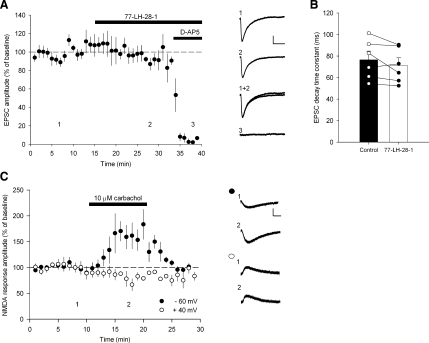
77-LH-28-1 Does Not Alter the Amplitude or Decay of Isolated NMDA EPSCs (A) Bath application of 77-LH-28-1 (10 μM) did not alter the amplitude of isolated NMDA receptor-mediated EPSCs, which were completely blocked by the bath application of D-AP5 (50 μM). Right: Example of current traces illustrating the mean EPSC response during the baseline (1), during the application of 77-LH-28-1 (2), and in the presence of D-AP5 (3). The scale bars represent 40 pA, 100 ms. (B) Bar graph showing the mean decay time constant of the NMDA receptor-mediated EPSC during the baseline and in the presence of 77-LH-28-1. The bar graph is overlaid with data from individual experiments. (C) Bath application of carbachol (10 μM) increased NMDA responses to exogenous NMDA application (1 mM) at −60 mV but not at +40 mV holding potential. Right: Example of traces of NMDA responses before (1) and after (2) carbachol application at the two membrane potentials. The scale bars represent 50 pA, 2 s. The data are plotted as the mean ± SEM.

Reports in the literature indicate NMDAR function is modulated by mAChRs (Aramakis et al., 1999; Harvey et al., 1993; Marino et al., 1998; Markram and Segal, 1990, 1992). This suggests that enhancement of NMDAR activation by mAChRs could be the mechanism for the facilitation of LTP by 77-LH-28-1. We tested this directly by recording NMDAR-mediated EPSCs in voltage clamp at a holding potential of −60 mV in the presence of NBQX (10 μM) and with reduced Mg^2+^ concentration (0.5 mM). Application of 10 μM 77-LH-28-1 produced no change in the amplitude (Figure 4A; 93% ± 6%, n = 6) or kinetics (Figure 4B; decay time constant 76 ± 8 ms control versus 71 ± 7 ms 77-LH-28-1, n = 6) of the NMDAR-mediated EPSC, and, therefore, we conclude there is no direct effect of M_1_ receptor activation on NMDARs.

To verify this result, we also measured NMDAR activity by exogenous NMDA application, an approach that has previously shown an enhancement of NMDAR function by mAChR activation (Aramakis et al., 1999; Harvey et al., 1993; Marino et al., 1998; Markram and Segal, 1990, 1992). We applied 1 mM NMDA by pressure application through a patch pipette placed in stratum radiatum close to the pyramidal cell-body layer while holding the CA1 pyramidal cell at −60 mV and with 1.3 mM Mg^2+^ in the artificial cerebrospinal fluid (aCSF). This produced stable responses that lasted ∼10 s, and under these conditions, bath application of 10 μM carbachol produced an increase in the amplitude of the NMDA response (Figure 4C; 150% ± 13%, n = 6, p < 0.05) similar to that seen in previous reports (Marino et al., 1998). However, when the membrane potential was set at +40 mV, the effect of carbachol application on NMDA responses was abolished (Figure 4C; 91% ± 4%, n = 5, p > 0.05). Taken together, these data indicate that NMDAR activation is enhanced by mAChR activation under conditions where Mg^2+^ block of NMDARs is present (Figure 4C; −60 mV) but not when Mg^2+^ is absent (Figure 4C; +40 mV) or significantly reduced (Figures 4A and 4B). This indicates that mAChR activation modulates synaptic NMDAR function via changes in membrane properties.

### M_1_ Receptors Enhance the NMDAR-Mediated Component of EPSPs

**Figure 5:**
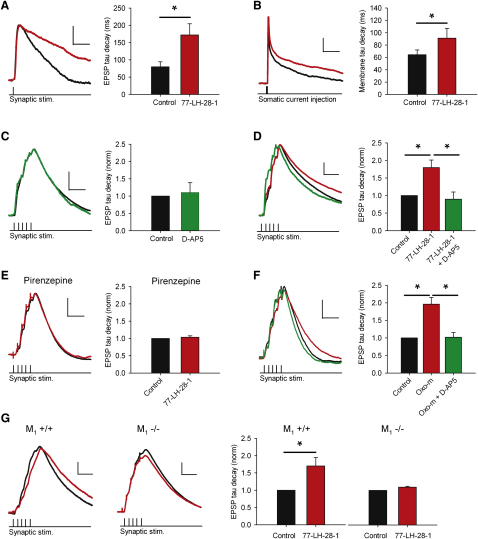
M_1_ Receptor Activation Enhances the NMDAR-Mediated Component of EPSPs (A) 77-LH-28-1 (10 μM) prolonged the duration of single EPSPs. Left: Example of voltage traces in the presence of 77-LH-28-1 (red) or control conditions (black). The scale bars represent 0.6 mV, 40 ms. Right: Average decay time constant increase. (B) 77-LH-28-1 (10 μM) prolonged the membrane decay time constant in response to a short subthreshold current injection. Left: Example of voltage traces in the presence of 77-LH-28-1 (red) or control conditions (black). The scale bars represent 0.6 mV, 20 ms. Right: Average membrane decay time constant increase. (C) Summated EPSPs during synaptic theta burst stimulation do not exhibit an NMDAR-mediated component. Left: Example of voltage traces of a burst of five EPSPs under control conditions (black) and in the presence of 50 μM D-AP5 (green). The scale bars represent 1.5 mV, 40 ms. Right: The average normalized decay time constant for a burst of five EPSPs does not change in the presence of D-AP5. (D) 77-LH-28-1 enabled NMDAR activation during synaptic theta burst stimulation. Left: Example of voltage traces showing a burst of five EPSPs under control conditions (black), in the presence of 10 μM 77-LH-28-1 (red), and after addition of 50 μM D-AP5 (green). The scale bars represent 2 mV, 40 ms. Right: The average normalized decay time constant is significantly reduced by addition of D-AP5 in the presence of 77-LH-28-1. (E) 77-LH-28-1 had no effect on EPSP duration in the presence of pirenzepine. Left: Example of voltage traces showing a burst of five EPSPs under control conditions (black) and in the presence of 10 μM 77-LH-28-1 (red) all in the presence of pirenzepine (25 μM). The scale bars represent 2 mV, 40 ms. Right: The average normalized decay time constant is not increased by 77-LH-28-1 in the presence of pirenzepine. (F) Oxotremorine-m enabled NMDAR activation during synaptic theta burst stimulation. Left: Example of voltage traces showing a burst of five EPSPs under control conditions (black), in the presence of 10 μM oxotremorine-m (red), and after addition of 50 μM D-AP5 (green). The scale bars represent 2 mV, 40 ms. Right: The average normalized decay time constant is significantly reduced by addition of D-AP5 in the presence of oxotremorine-m (oxo-m). (G) 77-LH-28-1 had no effect on EPSP duration in slices taken from M_1_−/− mice. Left: Example of voltage traces showing a burst of five EPSPs under control conditions (black) and in the presence of 10 μM 77-LH-28-1 (red) in M_1_+/+ and M_1_−/− mice. The scale bars represent 2 mV, 40 ms. Right: The average normalized decay time constant is increased in the presence of 77-LH-28-1 in M_1_+/+ mice but not M_1_−/− mice. ^∗^ indicates significant difference (p < 0.05). The data are plotted as the mean ± SEM.

We next recorded cells in current clamp and examined the EPSP waveform in the presence of the GABA_A_ receptor antagonist picrotoxin (50 μM) and the GABA_B_ receptor antagonist CGP55845 (1 μM). Under these conditions, application of 77-LH-28-1 prolonged the duration of EPSPs (Figure 5A) and increased the decay time constant (Figure 5A; 80 ± 15 ms in control, 172 ± 32 ms in 77-LH-28-1, n = 7, p < 0.05). The membrane time constant of the cell, measured by short subthreshold current injections, was also increased by application of 77-LH-28-1 (Figure 5B; 64 ± 8 ms in control, 91 ± 16 ms in 77-LH-28-1, n = 7, p < 0.05), which could, at least partially, account for the prolongation of the EPSP.

The depolarization and increase in input resistance caused by the activation of M_1_ receptors could also enhance NMDAR activation during synaptic transmission and in particular during TBP. To assess the component of the EPSP mediated by NMDARs during TBP, we gave five presynaptic stimuli at 100 Hz and compared the resulting EPSP waveform in the presence and absence of 50 μM D-AP5 (Figure 5C). EPSP amplitude was set to ensure that EPSP summation was of a similar magnitude to that used in the experiments shown in Figures 2 and 3. Under these control conditions, no NMDAR-mediated component of the EPSP could be detected (Figure 5C; decay time constant normalized to control, 1.1 ± 0.3, n = 8, p > 0.05). Application of 77-LH-28-1 depolarized the membrane potential by 2.8 ± 0.6 mV and caused a prolongation of the EPSPs similar to that seen in response to a single presynaptic stimulation (Figure 5D; decay time constant normalized to control, 1.8 ± 0.2, n = 6, p < 0.05). Under these conditions, D-AP5 reversed the EPSP prolongation induced by 77-LH-28-1 (Figure 5D; decay time constant normalized to control, 0.9 ± 0.2, n = 6, p < 0.05). When the membrane potential was repolarized to the membrane potential prior to 77-LH-28-1, D-AP5 still reduced the EPSP decay constant, indicating the EPSP prolongation and enhancement of the NMDAR-mediated EPSP was not due to membrane depolarization (decay constant normalized to control 1.1 ± 0.1 in 77-LH-28-1 and 0.77 ± 0.06 with addition of D-AP5, n = 6, p < 0.05).

The role of the M_1_ receptor in mediating the effects of 77-LH-28-1 on the NMDAR-mediated component of the EPSP was confirmed with pirenzepine to block the 77-LH-28-1-induced prolongation of EPSPs (Figure 5E; decay time constant normalized to control, 1.0 ± 0.1, n = 6, p > 0.05) and by using an orthosteric muscarinic receptor agonist oxotremorine-m (oxo-m, 10 μM) that produced a similar prolongation of the EPSP to 77-LH-28-1 and was also reversed by subsequent application of D-AP5 (Figure 5F; decay constant normalized to control 2.0 ± 0.2 in oxo-m and 1.0 ± 0.1 with addition of D-AP5, n = 6, p < 0.05). These results were further confirmed with mice lacking the M_1_ receptor. Application of 10 μM 77-LH-28-1 to slices taken from M_1_+/+ mice produced a prolongation of the EPSP similar to that found in slices from rats (Figure 5G; 1.7 ± 0.2, n = 5). In slices taken from M_1_−/− mice, the prolongation was absent (Figure 5G; 1.1 ± 0.03, n = 7). This indicates that the M_1_ receptor agonist 77-LH-28-1 specifically activates M_1_ receptors and augments the NMDAR-mediated component of the EPSP during the induction of LTP by TBP.

### M_1_ Receptors Enhance NMDAR Activation by Inhibition of SK Channels

**Figure 6:**
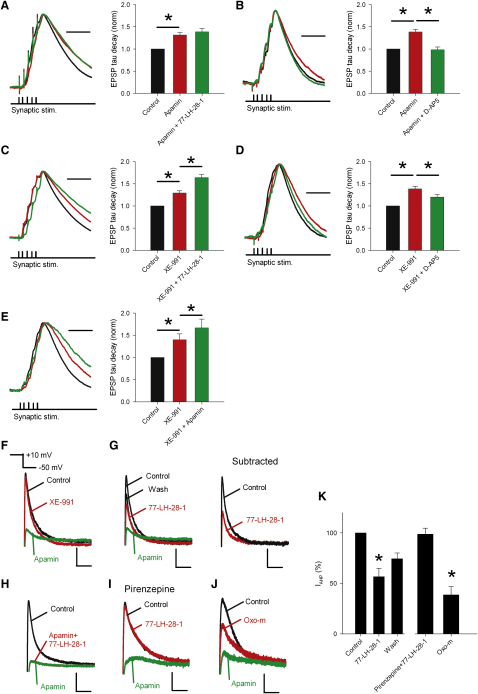
The M_1_ Receptor Agonist 77-LH-28-1 Prolongs the NMDAR-Mediated Component of EPSPs via Inhibition of SK Channels (A) Apamin prolongs the duration of summated EPSPs and occludes the action of 77-LH-28-1. Example of peak normalized voltage traces showing a burst of five EPSPs under control conditions (black), in the presence of 100 nM apamin (red), and after addition of 10 μM 77-LH-28-1 (green). The scale bar represents 40 ms. The average normalized decay time constant is significantly prolonged by addition of apamin with no further change in the presence of 77-LH-28-1. (B) Prolongation of EPSPs by apamin is reversed by application of D-AP5. Example of peak normalized voltage traces showing a burst of five EPSPs under control conditions (black), in the presence of 100 nM apamin (red), and after addition of 50 μM D-AP5 (green). The scale bar represents 40 ms. The average normalized decay time constant is significantly prolonged by addition of apamin and reversed by D-AP5. (C) The KCNQ channel blocker XE-991 prolongs the duration of summated EPSPs but does not occlude the action of 77-LH-28-1. Example of peak normalized voltage traces showing a burst of five EPSPs under control conditions (black), in the presence of 10 μM XE-991 (red), and after addition of 10 μM 77-LH-28-1 (green). The scale bar represents 40 ms. The average normalized decay time constant is significantly prolonged by addition of XE-991, but there is an additional significant prolongation with 77-LH-28-1. (D) Prolongation of EPSPs by XE-991 is partially reversed by application of D-AP5. Example of peak normalized voltage traces showing a burst of five EPSPs under control conditions (black), in the presence of 10 μM XE-991 (red), and after addition of 50 μM D-AP5 (green). The scale bar represents 40 ms. The average normalized decay time constant is significantly prolonged by addition of XE-991 and partially reversed by D-AP5. (E) Apamin prolongs the duration of summated EPSPs but does not occlude the action of XE-991. Example of peak normalized voltage traces showing a burst of five EPSPs under control conditions (black), in the presence of 100 nM apamin (red), and after addition of 10 μM XE-991 (green). The scale bar represents 40 ms. The average normalized decay time constant is significantly prolonged by addition of apamin, but there is additional significant prolongation with XE-991. (F) I_AHP_s recorded from CA1 pyramidal cells in the perforated patch configuration are primarily composed of current through SK channels. I_AHP_s are stimulated in the presence of TTX (1 μM) by switching the membrane potential from −50 mV to +10 mV for 100 ms. The I_AHP_ is seen after return to −50 mV. Application of XE-991 (10 μM) reduced I_AHP_ by 10% ± 2%; apamin (100 nM) blocked the remainder. The scale bars represent 50 pA, 50 ms. (G) 77-LH-28-1 (10 μM) inhibited the SK channel-mediated component of the I_AHP_ that partially recovered on washout. Subtraction of the apamin-insensitive component of the I_AHP_ revealed the SK-channel-mediated component (right). The scale bars represent 50 pA, 50 ms. (H) 77-LH-28-1 had no effect on I_AHP_ after application of 100 nM apamin. The scale bars represent 75 pA, 50 ms. (I) 77-LH-28-1 had no effect on I_AHP_ after incubation in 25 μM pirenzepine. The scale bars represent 30 pA, 50 ms. (J) Oxotremorine-m (oxo-m, 10 μM) inhibited the SK channel-mediated component of the I_AHP_. The scale bars represent 30 pA, 50 ms. (K) Summary of the effects of M_1_ receptor activation on I_AHP_ measured by pharmacological subtraction after apamin application. ^∗^ denotes statistical significance (p < 0.05). The data are plotted as the mean ± SEM.

Previous reports indicate that inhibition of SK channels prolongs the NMDAR-mediated component of EPSPs (Faber, 2010; Ngo-Anh et al., 2005) and that mAChRs can modulate SK channel function (Fiorillo and Williams, 2000; Gulledge and Stuart, 2005). Therefore, we tested whether the effect of 77-LH-28-1 on NMDAR activity was mediated by SK channels. Application of the selective SK channel blocker apamin (100 nM) caused a prolongation of summated EPSPs (Figure 6A; decay time constant normalized to control, 1.3 ± 0.1, n = 13, p < 0.01). Addition of 77-LH-28-1 did not produce any further prolongation of EPSPs (Figure 6A; decay time constant normalized to control, 1.4 ± 0.1, n = 13, p > 0.05 compared to apamin alone), indicating that apamin had occluded the action of 77-LH-28-1. Apamin application alone had no effect on either the membrane potential or input resistance of CA1 pyramidal cells (0 ± 0.2 mV and 28 ± 5 MΩ, p > 0.05, n = 20) and the apamin-induced EPSP prolongation was also completely reversed by subsequent application of D-AP5 (Figure 6B; decay time constant normalized to control, 1.39 ± 0.06 in apamin and 0.98 ± 0.06 with addition of D-AP5, n = 7, p < 0.05). Interestingly, contrary to previous reports, apamin produced no reliable effect on summated EPSP amplitude (100% ± 6%, n = 20) (Faber et al., 2005; Ngo-Anh et al., 2005). In addition, similar to the effects of 77-LH-28-1, apamin did not produce any change in EPSC amplitude or decay time constant (Figure S2).

M_1_ receptors are also known to modulate other potassium channels, classically inhibiting KCNQ channels, which are believed to underlie the M current (Marrion et al., 1989). When we tested the effects of the KCNQ channel blocker XE-991 (10 μM), the summated EPSPs were prolonged in a similar fashion to apamin (Figure 6C; decay time constant normalized to control, 1.3 ± 0.1, n = 13, p < 0.01). However, subsequent addition of 77-LH-28-1 produced a significant additional prolongation (Figure 6C; decay time constant normalized to control, 1.6 ± 0.1, n = 13, p < 0.05 compared to XE-991 alone), indicating that XE-991 does not occlude the action of 77-LH-28-1. XE-991 application alone produced a depolarization of the membrane potential and an increase in the input resistance of CA1 pyramidal cells (1.7 ± 0.4 mV and 47 ± 5 MΩ, p < 0.05, n = 18) and the XE-991-induced EPSP prolongation was only partially reversed by subsequent application of D-AP5 (Figure 6D; decay time constant normalized to control, 1.4 ± 0.1 in apamin and 1.2 ± 0.1 with addition of D-AP5, n = 7, p < 0.05 for control versus XE-991 and XE-991 versus D-AP5).

The effects of KCNQ channel and SK channel blockade on EPSP prolongation could be further separated into two distinct processes. The application of apamin produced an additional prolongation of the EPSP after the initial prolongation by XE-991 (Figure 6E; decay time constant normalized to control, 1.40 ± 0.13 for XE-991 and 1.66 ± 0.20 for apamin combined with XE-991, n = 11, p < 0.05 for control versus XE-991 and XE-991 versus apamin). These data indicate that membrane depolarization and increased input resistance seen in the presence of XE-991 are not sufficient to occlude the effects of M_1_ receptor activation on NMDAR function.

Muscarinic receptors have previously been shown to transiently enhance SK channel function by causing release of Ca^2+^ from internal stores (Gulledge and Stuart, 2005), but there is no evidence for muscarinic receptor activation inhibiting SK channel function. Therefore, we sought to confirm that M_1_ receptors inhibit SK channels by measuring SK channel current directly. To do this, we recorded from CA1 pyramidal cells in voltage clamp using the perforated-patch technique. Depolarization of the cell from −50 mV to +10 mV for 100 ms and back to −50 mV revealed an afterhyperpolarization current (I_AHP_) that was largely insensitive to the KCNQ channel blocker XE-991 (10 μM, 90% ± 2% of peak control current, n = 6) but was robustly inhibited by the SK channel blocker apamin (100 nM, 10% ± 2% of peak control current, n = 6) (Figure 6F). For all subsequent experiments, XE-991 (10 μM) was present throughout and the SK channel-mediated component of the I_AHP_ was calculated by subtraction of the current remaining in apamin. The M_1_ receptor agonist 77-LH-28-1 reduced the SK channel-mediated component of the I_AHP_ to 57% ± 8% (Figures 6G and 6K; n = 6, p < 0.05). When 77-LH-28-1 was removed, the inhibition partially reversed (74% ± 6%). We confirmed that activation of M_1_ receptors inhibits the SK channel-mediated component of I_AHP_ by reversing the sequence of drug application with the result that 77-LH-28-1 produced no additional inhibition of I_AHP_ when applied after apamin (Figures 6H and 6K). The specificity of 77-LH-28-1 was confirmed by preincubation with pirenzipine (25 μM), which blocked the inhibition of I_AHP_ by 77-LH-28-1 (Figures 6I and 6K; 99% ± 6% of control, n = 5, p > 0.05) and by the use of oxotremorine-m (oxo-m, 10 μM), which produced a similar inhibition of I_AHP_ to 77-LH-28-1 (Figures 6J and 6K; 39% ± 8% of control, n = 6, p < 0.05).

### Inhibition of PKC Blocks the Inhibition of SK Channels by M_1_ Receptors

**Figure 7:**
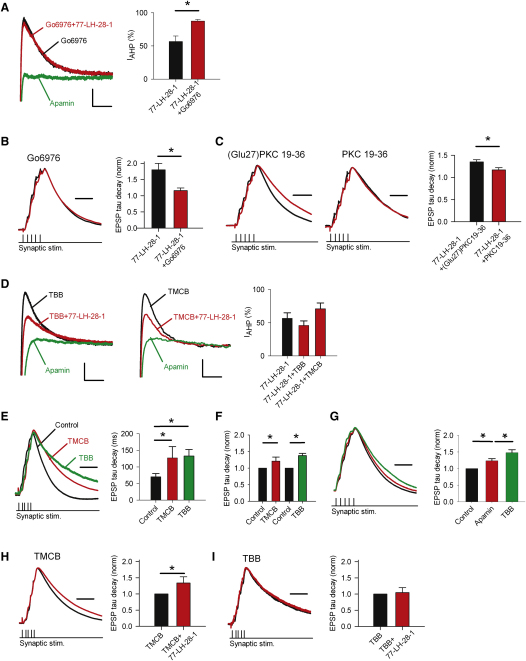
PKC Mediates the M_1_ Receptor-Induced Inhibition of SK Channels (A) Inhibition of I_AHP_ by 77-LH-28-1 (10 μM) is reduced by preincubation with the PKC inhibitor Go6976 (100 nM). The scale bars represent 30 pA, 50 ms. (B) Prolongation of EPSPs by 77-LH-28-1 is blocked by preincubation with the PKC inhibitor Go6976 (100 nM). Example of peak normalized voltage traces showing a burst of five EPSPs after preincubation with Go6976 (black) and no prolongation in the presence of 10 μM 77-LH-28-1 (red). The scale bar represents 40 ms. The average normalized decay time constant is not significantly prolonged. (C) Prolongation of EPSPs by 77-LH-28-1 is blocked by inclusion of the PKC inhibitor PKC 19-36 in the patch pipette. Example of peak normalized voltage traces showing a burst of five EPSPs after infusion of (Glu27)PKC 19-36 or PKC 19-36 (black). Prolongation in the presence of 10 μM 77-LH-28-1 (red) only occurs in the presence of (Glu27)PKC 19-36. The scale bar represents 40 ms. The average normalized decay time constant is significantly prolonged by 77-LH-28-1 in the presence of (Glu27)PKC 19-36 and this is significantly reduced by PKC 19-36. (D) Inhibition of I_AHP_ by 77-LH-28-1 (10 μM) is unchanged by preincubation with the CK2 inhibitors TBB (10 μM) or TMCB (10 μM). The scale bars represent 30 pA, 50 ms. (E) Incubation in TMCB (10 μM) or TBB (10 μM) greatly prolonged the EPSP compared to control. Example of peak normalized voltage traces showing a burst of five EPSPs under control conditions (black) and, in separate experiments, after incubation in 10 μM TMCB (red) or 10 μM TBB (green). The scale bar represents 60 ms. The average decay time constant is significantly prolonged by incubation in TMCB or TBB. Input resistance after incubation in TMCB or TBB was unchanged compared to control. (F) Acute application of TMCB (10 μM) or TBB (10 μM) prolonged the average normalized decay time constant of the EPSP. (G) Apamin prolongs the duration of summated EPSPs but does not occlude the action of TBB. Example of peak normalized voltage traces showing a burst of five EPSPs under control conditions (black), in the presence of 100 nM apamin (red), and after addition of 10 μM TBB (green). The scale bar represents 40 ms. The average normalized decay time constant is significantly prolonged by addition of apamin, but there is an additional significant prolongation with 30 min application of TBB. (H) Incubation in TMCB does not block the action of 77-LH-28-1. Example of peak normalized voltage traces showing a burst of five EPSPs after incubation in TMCB (black) and with addition of 10 μM 77-LH-28-1 (red). The scale bar represents 60 ms. The average normalized decay time constant shows a significant change in the presence of 77-LH-28-1. (I) Incubation in TBB blocks the action of 77-LH-28-1. Example of peak normalized voltage traces showing a burst of five EPSPs after incubation in TBB (black) and after addition of 10 μM 77-LH-28-1 (red). The scale bar represents 60 ms. The average normalized decay time constant shows no change in the presence of 77-LH-28-1. The data are plotted as the mean ± SEM.

We next investigated the mechanism for the M_1_ receptor-mediated inhibition of SK channels. M_1_ receptors are thought to couple to Gq/11 subunits whose downstream signaling bifurcates into the production of IP_3_ and DAG, leading to activation of PKC (Delmas and Brown, 2005). Therefore, we tested whether PKC inhibitors could block the M_1_ receptor-mediated prolongation of EPSPs and inhibition of I_AHP_. Bath application of the PKC inhibitor Go6976 (200 nM) significantly reduced the M_1_ receptor-induced inhibition of I_AHP_ (Figure 7A; 87% ± 2% of control, n = 6, p < 0.05 compared to 77-LH-28-1), indicating that activation of PKC by M_1_ receptors is necessary for the inhibition of SK channels. Go6976 also significantly reduced the prolongation of EPSPs induced by application of 77-LH-28-1 (Figure 7B; decay time constant normalized to control, 1.16 ± 0.08, n = 6, p < 0.05 compared to 77-LH-28-1). Similarly, inclusion of the inhibitory PKC fragment PKC 19-36 in the patch pipette also significantly reduced the prolongation of EPSPs induced by application of 77-LH-28-1 compared to the prolongation induced in the presence of the inactive single mutation control peptide (Glu27)PKC 19-36 (House and Kemp, 1987) (Figure 7C; decay time constant normalized to control, 1.35 ± 0.05, n = 7 versus 1.17 ± 0.05, n = 14, p < 0.05 for (Glu27)PKC 19-36 versus PKC 19-36, respectively).

The calcium sensitivity of SK channels has been shown to be regulated by casein kinase 2 (CK2) in response to neuromodulators such as noradrenaline (Allen et al., 2007; Maingret et al., 2008). Therefore, we also tested whether the CK2 inhibitors 4,5,6,7-tetrabromobenzotriazole (TBB) or 2-(4,5,6,7-tetrabromo-2-(dimethylamino)-1H-benzo[d]imidazol-1-yl) acetic acid (TMCB) (Pagano et al., 2008) could block the M_1_ receptor-induced inhibition of I_AHP_. Incubation of slices in either 10 μM TBB or 10 μM TMCB for at least 1 hr had no effect on the M_1_ receptor-induced inhibition of I_AHP_ (Figure 7D; TBB, 46% ± 7% of control, n = 6; TMCB, 71% ± 9% of control, p > 0.05 compared to 77-LH-28-1 for both) suggesting that CK2 activity is not required for the inhibition of SK channels. CK2 also has direct effects on NMDAR function (Lieberman and Mody, 1999; Sanz-Clemente et al., 2010). We found that incubation in 10 μM TMCB or TBB for periods of more than 1 hr greatly prolonged EPSPs (Figure 7E; decay time constant, TMCB, 127 ± 34 ms, n = 9; TBB, 156 ± 29 ms, p < 0.05 compared to control for both). This prolongation was also seen after 20 min of acute TMCB or TBB application (Figure 7F; decay time constant normalized to control, TMCB, 1.21 ± 0.07, n = 7, TBB, 1.38 ± 0.12, n = 7, p < 0.05 for both) but this prolongation was still seen in the presence of apamin (Figure 7G; decay time constant normalized to control, apamin 1.24 ± 0.06, TBB 1.43 ± 0.09, n = 6, p < 0.05), indicating that the effects of CK2 inhibition on EPSP duration are not mediated by SK channels. Application of 77-LH-28-1 produced an additional prolongation of the EPSP after incubation in TMCB (Figure 7H; decay time constant normalized to TMCB, 1.34 ± 0.07, n = 9, p < 0.05) but not TBB (Figure 7I; decay time constant normalized to TBB, 1.05 ± 0.15, n = 6, p > 0.05). However, the large apamin-insensitive prolongation of EPSPs by CK2 inhibition precluded strong conclusions being drawn concerning the role of CK2 in M_1_-induced prolongation of EPSPs.

### Inhibition of SK Channels by Apamin Facilitates LTP Induction

**Figure 8:**
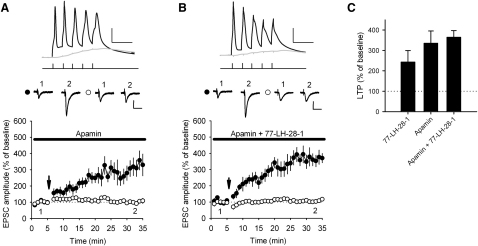
SK Channel Inhibition Facilitates LTP Induction (A) SK channel blockade facilitates LTP induction. In the continuous presence of apamin (100 nM) TBP induces pathway-specific LTP. The arrow indicates the timing of the TBP protocol. Top: Example of voltage traces from a single experiment showing the initial burst of five coincident EPSPs and action potentials (black) and a single test burst of five subthreshold EPSPs (gray). The scale bars represent 25 mV, 20 ms. Middle: Example of current traces from a single experiment illustrating the mean EPSC response during the baseline (1) and at 30–35 min (2) in the test (black circles) and control (white circles) pathways. The scale bars represent 50 pA, 40 ms. (B) In the continuous presence of apamin (100 nM) and 77-LH-28-1 (10 μM), TBP induces pathway-specific LTP similar in magnitude to apamin or 77-LH-28-1 in isolation. The arrow indicates the timing of the TBP protocol. Symbols as described in (A). Top: Example of voltage traces. The scale bars represent 25 mV, 20 ms. Middle: Example of EPSC current traces from a single experiment. The scale bars represent 50 pA, 40 ms. (C) 77-LH-28-1 (10 μM), apamin (100 nM), or a combination of 77-LH-28-1 and apamin all induce a similar amount of LTP. The data are plotted as the mean ± SEM.

The application of apamin has previously been shown to facilitate the induction of LTP (Behnisch and Reymann, 1998; Lin et al., 2008; Ngo-Anh et al., 2005; Stackman et al., 2002) and genetic overexpression of SK2 channels inhibits the induction of LTP (Hammond et al., 2006). We confirmed the role of SK channels in the facilitation of LTP by applying TBP in the presence of 100 nM apamin, which produced significant pathway-specific LTP (Figure 8A; 100% ± 10% control versus 335% ± 60% test pathway, n = 8, p < 0.01). Furthermore, a similar magnitude LTP was induced in the continuous presence of both 10 μM 77-LH-28-1 and 100 nM apamin (Figure 8B; 107% ± 5% control versus 365% ± 33% test pathway, n = 7, p < 0.01), indicating that no additional facilitation of LTP was gained by stimulation of M_1_ receptors after blockade of SK channels (Figure 8C).

## Discussion

Activation of mAChRs facilitates the induction of LTP in the hippocampus (Boddeke et al., 1992; Ovsepian et al., 2004; Shinoe et al., 2005) and is critical for various forms of learning and memory (Atri et al., 2004; De Rosa and Hasselmo, 2000; Warburton et al., 2003). The present study confirms a critical role for M_1_ receptors in the facilitation of LTP and provides data demonstrating a role for SK channels mediating this facilitation.

mAChR activation has been linked to modulation of potassium channels such as M-channels (KCNQ) (Pfaffinger et al., 1985; Seeger and Alzheimer, 2001) and G protein-coupled inward-rectifier potassium channels (GIRK) (Brown et al., 1997). Our data support an additional inhibition of SK channels that is critical for the facilitation of LTP. SK channels have been shown to be ideally placed on the postsynaptic spine of Schaffer collateral synapses in the hippocampus where they can act to rapidly repolarize dendritic spines after AMPA receptor activation thereby limiting NMDAR activation and calcium influx through voltage-activated calcium channels (Bloodgood and Sabatini, 2007; Lin et al., 2008; Ngo-Anh et al., 2005). Studies have also shown that blocking SK channels facilitates the induction of LTP (Behnisch and Reymann, 1998; Stackman et al., 2002) and that genetic overexpression of SK2 channels inhibits LTP induction and hippocampal-dependent learning (Hammond et al., 2006). However, until now it was unclear whether SK channel function could be modulated to regulate the induction of LTP. In the present study, we show that SK channels mediating a component of the I_AHP_ are directly modulated by M_1_ receptors (Figure 6) and that blockade of SK channels with apamin occludes the action of M_1_ receptor activation on NMDAR function (Figure 6) and LTP induction (Figure 8). Therefore, our data support the conclusion that SK channels can regulate the induction of LTP and provide evidence that SK channel function is controlled by activation of M_1_ receptors.

The detailed mechanism and the molecular interactions that connect M_1_ receptor activation to SK channel inhibition are currently unknown. It has been shown that M_1_ receptors and the G protein subunits Gq/11 are located at dendritic spines (Tanaka et al., 2000; Yamasaki et al., 2010). Gq/11 subunits' downstream signaling bifurcates into the production of IP_3_ and DAG, which has been shown to promote Ca^2+^ release from intracellular stores, activate PKC, and locally deplete PIP_2_ levels (Delmas and Brown, 2005). Our data support the view that PKC activation is necessary for the inhibition of SK channels, but we cannot rule out an additional role for Ca^2+^ release and/or PIP_2_ depletion. Interestingly, SK channel inhibition by noradrenergic receptors in superior cervical ganglion cells acts through a different mechanism involving CK2 rather than PKC leading to a decrease in the calcium sensitivity of SK channels (Maingret et al., 2008). However, there is little evidence that M_1_ receptors couple to CK2. Our results suggest that muscarinic receptor inhibition of SK channels is not mediated by CK2. There is emerging evidence demonstrating that CK2 directly modulates NMDAR (Lieberman and Mody, 1999) or regulates NMDAR subunit complement (Sanz-Clemente et al., 2010), and our results showing a SK channel-independent modulation of EPSP duration (Figure 7) potentially support such roles for CK2 at Schaffer collateral synapses in the hippocampus.

Although KCNQ channels are thought to be preferentially targeted to the somatic membrane and are not present on dendritic spines, their blockade may still increase NMDAR activation as a result of cellular membrane depolarization and increase in input resistance (Hu et al., 2007; Yue and Yaari, 2004). While this may indeed be the case, our data indicate that KCNQ channels do not mediate the facilitation of NMDAR function induced by M_1_ receptor activation since blockade of KCNQ channels with XE-991 did not prevent the actions of M_1_ receptor activation on NMDAR function (Figure 6). The actions of M_1_ receptors may therefore be separated into (1) an inhibition of SK channels that promotes NMDAR activity in dendritic spines and (2) an inhibition of KCNQ channels at the somatic membrane that depolarizes the membrane potential and increases input resistance. The other ion channels thought to be modulated by mAChRs, GIRK channels, are not thought to be modulated by M_1_ receptors in CA1 pyramidal cells and are instead activated by M_2_ receptors (Seeger and Alzheimer, 2001). Our results also show that M_1_ receptors inhibit somatic SK channels, which will presumably increase excitability by reducing the AHP (Figure 6).

Our data show that M_1_ receptor activation prolongs the NMDAR-mediated component of the EPSP during LTP induction, and we suggest this is the mechanism for the facilitation of LTP (Figure 5). We also show that under optimized voltage clamp conditions, such as recording NMDA responses at +40 mV or small NMDAR-mediated EPSCs at −60 mV in reduced Mg^2+^, NMDAR activity is unchanged by M_1_ receptor activation (Figure 4). These data argue strongly against a direct action of M_1_ receptor activation on NMDARs, such as has been suggested in previous reports (Aramakis et al., 1999; Harvey et al., 1993; Marino et al., 1998; Markram and Segal, 1990, 1992). However, because of the voltage-dependent nature of the SK inhibition of NMDARs, when we record exogenous NMDA responses at −60 mV or EPSPs in current clamp, NMDAR activity is enhanced (Figure 4).

From a therapeutic point of view, this study suggests 77-LH-28-1 and similar compounds may be highly attractive as potential treatments for cognitive disorders (Dean et al., 2003). Currently, the only effective treatments for patients with Alzheimer's disease are cholinesterase inhibitors and memantine. M_1_ receptor agonists have been shown to ameliorate Aβ and tau pathology in animal models of Alzheimer's disease (Caccamo et al., 2006) and to be beneficial for patients suffering from Alzheimer's disease and schizophrenia (Dean et al., 2003; Koch et al., 2005) so the development of new allosteric M_1_ receptor agonists could provide a major breakthrough in the treatment of these cognitive disorders.

We have shown that LTP is facilitated when M_1_ receptors are activated by either the specific allosteric M_1_ receptor agonist 77-LH-28-1 (Figure 2) or the endogenous ligand acetylcholine (Figure 3). Since TBP does not induce LTP in control conditions, the facilitation by M_1_ receptor activation is seen as a gating of LTP. Interestingly, the same is true when LTP is induced by patterns of activity believed to occur in vivo during exploration (Isaac et al., 2009). Therefore, the regulation of SK channel function by M_1_ receptor activation may be a critical step in the induction of hippocampal LTP in vivo.

## Experimental Procedures

### Slice Preparation

Brain slices were prepared from P13-15 male Wistar rats or P60-70 male C57BL/6J mice lacking M_1_ receptors (M_1_−/− kindly provided by Dr. J. Wess [Miyakawa et al., 2001]) or age matched wild-type mice (M_1_+/+). Following a lethal dose of anesthetic (isoflurane inhalation), brains were removed and dissected in ice-cold aCSF (in mM, 119 NaCl, 2.5 KCl, 1 NaH_2_PO_4_.H_2_O, 26.2 NaHCO_3_, 10 glucose, 2.5 CaCl_2_, and 1.3 MgSO_4_) saturated with 95% O_2_ and 5% CO_2_. Parasagittal hippocampal slices 300–400 μm thick were cut with a vibratome (DTK-1000, DSK, Japan, or VT1200, Leica, Germany) and slices were incubated in aCSF at 36°C for 30 min and then stored at room temperature until use. Before being transferred to the submerged recording chamber, the connections between CA3 and CA1 were cut. All experiments were performed in accordance with Home Office guidelines as directed by the Home Office Licensing Team at the University of Bristol.

### Whole-Cell Patch Clamp Recording

Slices were placed in a submerged recording chamber perfused with aCSF (as above) at room temperature with the addition of 50 μM picrotoxin. CA1 pyramidal cells were visualized with infrared DIC optics on an Olympus BX-50 microscope. Patch electrodes with a resistance of 4–5 MΩ were pulled from borosillicate filamented glass capillaries (Harvard Apparatus) with a vertical puller (PC-10, Narashige, Japan). Pipettes were filled with intracellular solution containing (in mM) 120 KMeSO_3_, 10 HEPES, 0.2 EGTA, 4 Mg-ATP, 0.3 Na-GTP, 8 NaCl, and 10 KCl and set to pH 7.4, 280–285 mOsm.

Recordings from CA1 pyramidal neurons were made with a multiclamp 700A amplifier (Molecular Devices, USA), filtered at 4 kHz and digitized at 10 kHz with a data acquisition board and Signal acquisition software (CED, Cambridge, UK). Cells were voltage clamped at −75 or −80 mV (after junction potential correction of −9.1 mV). Series resistance was monitored throughout the experiments and cells that showed >20% change were discarded from subsequent analysis. Recordings were also rejected from analysis if the series resistance was greater than 30 MΩ. Bridge balance was employed for all current clamp recordings.

Perforated-patch recordings were performed with patch pipettes of 4–5 MΩ resistance tip filled with the same intracellular solution to whole-cell recordings. Pipettes were then backfilled with the same intracellular solution supplemented with gramicidin (80 μg/ml). Gramicidin was prepared as a stock solution in DMSO (20 mg/ml). After formation of a gigaohm seal, the series resistance was monitored and recordings were commenced once stable. Series resistances averaged 36.1 ± 1.7 MΩ (n = 28), and recordings were not used if the series resistance changed by >20% during data collection. Spontaneous rupture of the patched membrane was checked by continuous monitoring of series resistance. Tetrodotoxin (TTX; 1 μM) was continuously present in the aCSF. No leak subtraction was employed and the components of the I_AHP_ mediated by KCNQ or SK channels were assessed pharmacologically.

Extracellular field potential recordings were made from hippocampal slices bathed in aCSF containing picrotoxin with a patch pipette filled with aCSF. The initial slope of evoked synaptic responses was measured to calculate the amplitude of responses.

Synaptic responses were evoked in control and test pathways with 100 μs square voltage steps applied at 0.1 Hz through two bipolar stimulating electrodes (FHC) located in stratum radiatum with the test pathway proximal and the control pathway distal to the pyramidal cell layer. Average baseline EPSC amplitudes in control and test pathways were similar for all LTP experiments. Postsynaptic action potentials were initiated through somatic current injections (2 nA, 2 ms) that reliably induced action potentials in all conditions.

The resting membrane potential and input resistance of the cell were monitored in current clamp for a stable baseline period of 10–20 min before cholinergic agonists were washed into the recording chamber. The membrane potential and input resistance were monitored for a further 10–20 min. Input resistances were measured after the membrane voltage reached steady state.

Focal application of 1 mM NMDA was performed through a glass electrode with a resistance of 3–4 MΩ placed in stratum radiatum close to the cell-body layer. A 1 mM NMDA solution in aCSF was pressure ejected (150 ms, 30–90 kPa) under the control of a spritzer (made in house). For the application of NMDA, 1 μM TTX was added to the aCSF and the intracellular solution contained (in mM) 117 CsMeSO_3_, 10 HEPES, 5 QX314-Cl, 0.2 EGTA, 4 Mg-ATP, 0.3 Na-GTP, and 8 NaCl and set to pH 7.4, 280–285 mOsm.

CGP55845, D-AP5, LY341495, NBQX, picrotoxin, pirenzepine, oxotremorine-m, TBB, (Glu27)PKC 19-36, and Go6976 were purchased from Tocris. Apamin, TTX, TMCB, and XE-991 were purchased from Ascent Scientific. PKC 19-36 was purchased from Sigma. 77-LH-28-1 was a gift from GlaxoSmithKline. PKC 19-36 and (Glu27)PKC 19-36 were infused for at least 30 min before application of 77-LH-28-1. Slices were incubated in TBB and TMCB for at least 1 hr before application of 77-LH-28-1.

### Induction of Synaptic Plasticity

EPSCs were recorded in voltage clamp from two independent pathways. TBP was applied after the neurons were switched into current clamp mode within 10 min of reaching the whole-cell configuration to prevent wash-out of plasticity. The TBP protocol consisted of a train of ten bursts where each burst consisted of five stimulations at 100 Hz with the frequency of bursts set at 5 Hz. Three trains were given separated by 10 s intervals. Where plasticity experiments were carried out in the presence of 77-LH-28-1, apamin, or XE-991, the drugs were washed into the bath before the whole-cell configuration was achieved and perfused throughout the experiment.

### Muscarinic EPSP

Experiments involving stimulation of the muscarinic EPSP were carried out in horizontal slices as this maximized the density and connectivity of cholinergic fibers in the stratum oriens. The effects of 77-LH-28-1 on LTP induction were qualitatively similar in horizontal versus parasagittal slices. The muscarinic EPSP was elicited by a high-frequency burst of stimulation (4 stimuli at 100 Hz) delivered to a bipolar stimulating electrode placed in stratum oriens. The stratum oriens stimulation was delivered 2 s before the TBP protocol to ensure the peak of the muscarinic EPSP coincided with the start of the TBP protocol.

### Data Analysis

Sweeps from the test and control pathways were separated and six consecutive traces were averaged together to produce a mean response every minute. EPSC amplitude measurements were taken from the mean traces and normalized to the mean baseline EPSC amplitude. Data are plotted as the mean ± standard error of the mean (SEM).

Statistical tests were performed with paired or unpaired Student's t tests as appropriate. LTP was assessed by comparing the mean normalized EPSC amplitudes (or fEPSP slopes) in control and test pathways 25–30 min after induction.

## Supplemental Information

Document S1. Two Figures
