# Facilitation of Long-Term Potentiation by Muscarinic M1 Receptors Is Mediated by Inhibition of SK Channels

**DOI:** 10.1016/j.neuron.2011.02.030

**Published:** 2011-03-10

**Authors:** Katherine A. Buchanan, Milos M. Petrovic, Sophie E.L. Chamberlain, Neil V. Marrion, Jack R. Mellor

(Neuron *68*, 948–963; December 8, 2010)

The original publication omitted one affiliation for Dr. M. Petrovic: Institute of Medical Physiology, School of Medicine, University of Belgrade, 11000 Belgrade, Serbia. This affiliation has been added to the article online.

