# Supplementary material for: Facilitation of Long-Term Potentiation by Muscarinic M1 Receptors Is Mediated by Inhibition of SK Channels
Source: Neuron. 2010 Dec 9;68(5-6):948–63. doi: 10.1016/j.neuron.2010.11.018 (PMC3003154; doi:10.1016/j.neuron.2010.11.018)
Supplement: Document S1. Two Figures [file mmc1.pdf]

Supplemental Information
Neuron, Volume *68*

Facilitation of Long-Term Potentiation by Muscarinic M_1 Receptors Is Mediated by Inhibition of SK Channels

Katherine A. Buchanan, Milos M. Petrovic, Sophie E. L. Chamberlain, Neil V. Marrion, and Jack R. Mellor

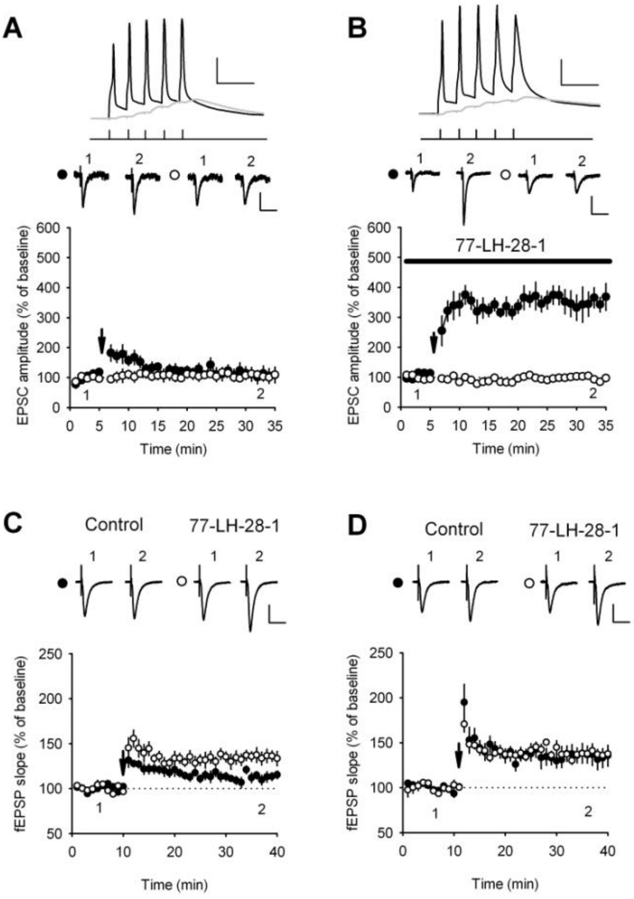

Figure S1. Related to Figure 2. Facilitation of LTP induction by M_1 receptor agonist 77-LH-28-1

(A) TBP does not induce LTP under control conditions at 35°C. Bottom, TBP induced no change in EPSC amplitude in the test (black circles) or control (white circles) pathways. The arrow indicates the timing of the TBP protocol. Top, example voltage traces from a single experiment showing the initial burst of 5 coincident EPSPs and action potentials (black) and a single test burst of 5 subthreshold EPSPs (grey). Scale bars 25 mV, 20 ms. Middle, example current traces from a single experiment illustrating the mean EPSC response during the baseline (1) and at 30-35 minutes (2) in the test and control pathways. Scale bars 30 pA, 40 ms.

(B) TBP induces LTP in the presence of 77-LH-28-1 at 35°C. Bottom, in the presence of 77-LH-28-1 (10 μM) TBP induced pathway specific LTP. Symbols as described in (A). Top, example voltage traces as described in (A). Scale bars 25 mV, 20 ms. Middle, example EPSC current traces from a single experiment as described in (A). Scale bars 30 pA, 40 ms.

(C) Using field potential recordings, a theta burst protocol consisting of 10 bursts at a frequency of 5 Hz where each burst consisted of 5 pulses at 100 Hz produced a small but significant LTP (black circles). In the presence of 77-LH-28-1 the amount of LTP was increased (white circles). Example current traces from a single experiment illustrating the mean EPSC response during the baseline (1) and at 30-35 minutes (2) in control and 77-LH-28-1. Scale bars 0.2 mV, 30 ms.

(D) A maximal LTP protocol consisting of the theta burst protocol repeated 3 times with an interval of 10 seconds induced significant LTP (black circles) that was similar in magnitude to that induced in the presence of 77-LH-28-1 (white circles). Example current traces from a single experiment illustrating the mean EPSC response during the baseline (1) and at 30-35 minutes (2) in control and 77-LH-28-1. Scale bars 0.2 mV, 30 ms.

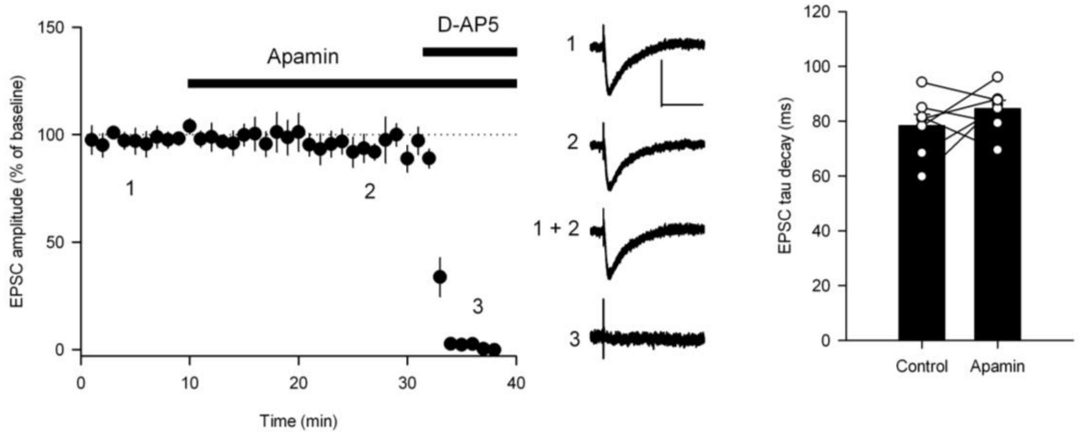

Figure S2. Related to Figure 6. The effects of apamin on NMDAR-mediated EPSCs
Apamin did not alter the amplitude of isolated NMDAR-mediated EPSCs which were completely blocked by the bath application of D-AP5 (50 µM). Right, example current traces illustrating the mean EPSC response during the baseline (1), application of apamin (2) and in the presence of D-AP5 (3). Scale bars 40 pA, 100 ms. Bar graph shows the mean decay time constant of the NMDAR-mediated EPSC during the baseline and in the presence of apamin.
